# Population Diversity of Antibiotic Resistant *Enterobacterales* in Samples From Wildlife Origin in Senegal: Identification of a Multidrug Resistance Transposon Carrying *bla*_*CTX*–*M*–15_ in *Escherichia coli*

**DOI:** 10.3389/fmicb.2022.838392

**Published:** 2022-03-18

**Authors:** Rim Abdallah, Edmond Kuete Yimagou, Linda Hadjadj, Oleg Mediannikov, Ahmad Ibrahim, Bernard Davoust, Amanda Barciela, R. Adriana Hernandez-Aguilar, Georges Diatta, Cheikh Sokhna, Didier Raoult, Jean-Marc Rolain, Sophie Alexandra Baron

**Affiliations:** ^1^IRD, APHM, MEPHI, Faculté de Médecine et de Pharmacie, Aix Marseille University, Marseille, France; ^2^IHU Méditerranée Infection, Marseille, France; ^3^Dindefelo Biological Station, Jane Goodall Institute Spain and Senegal, Kedougou, Senegal; ^4^Department of Social Psychology and Quantitative Psychology, Faculty of Psychology, Serra Hunter Programme, University of Barcelona, Barcelona, Spain; ^5^VITROME IRD 257, Campus International de Recherche IRD-UCAD de Hann, Dakar, Senegal

**Keywords:** antibiotic resistance, *Enterobacterales*, primates, environment, Senegal

## Abstract

**Introduction:**

The role of wildlife in the transmission of antimicrobial resistant (AMR) is suspected but scarcely reported in current studies. Therefore, we studied the dynamics and prevalence of antibiotic-resistant *Enterobacterales* in antibiotic-limited areas of Senegal.

**Materials and Methods:**

We collected fecal samples from monkeys and apes (N = 226) and non-fecal environmental samples (N = 113) from Senegal in 2015 and 2019. We grew the samples on selective media, subsequently isolated AMR *Enterobacterales*, and then sequenced their genomes.

**Results:**

We isolated 72 different *Enterobacterales* among which we obtained a resistance rate of 65% for colistin (N = 47/72) and 29% for third generation-cephalosporin (C3G) (29%, N = 21/72). Interestingly, almost 46% of our isolates, among *Enterobacter* sp., *Citrobacter cronae* and *Klebsiella aerogenes*, belong to 34 new STs. Moreover, the genes *bla*_*CTX*–*M*–15_, *bla*_*TEM*1*B*_, *sul2*, *dfrA14*, *qnrs*, *aph*(*3′′*), *aph*(*6*), *tetA*, and *tetR* harbored within a transposon on the *IncY* plasmid of ST224 *Escherichia coli* were transferred and inserted into a ST10 *E*. *coli* phage coding region.

**Conclusion:**

Wildlife constitutes a rich, unexplored reservoir of natural microbial diversity, AMR genes and international resistant clones pathogenic in humans. The presence of a transposon that carries AMR genes is intriguing since no antibiotics are used in the non-human primates we studied.

## Introduction

Antimicrobial resistant (AMR) bacteria are widely found in humans, animals, and the environment, which could be all part of the same ecosystem in certain areas (McEwen and Collignon, 2018). AMR genes can be transferred within this ecosystem, and homologous resistance genes could be found in pathogens, normal flora and soil bacteria (Woolhouse et al., 2015). AMR, even in a “One Health” perspective, is considered to be linked to human activity, in hospitals and livestock farms or the artificial environment created by people such as houses and cities. However, this often overlooks the role played by the wild environment, where human activity is lower than in artificial environments created by humans (Chokshi et al., 2019).

According to the World Health Organization (WHO), Africa has one of the largest gap in data on the incidence of AMR (World Health Organization, 2014) and genomic analysis of this AMR bacteria in African wildlife has rarely been explored, to our knowledge (Ramey and Ahlstrom, 2019; Miller et al., 2020). However, it is essential information for understanding the sources of antibiotic resistance, its spread and the factors involved in this spread. In Africa, and more precisely in Senegal, AMR has mainly been studied in relation to human medicine (Dromigny et al., 2005), as well as animal farming (Vounba et al., 2019). High levels of resistance in this country indicate that human hospital outbreaks are reservoirs of extended spectrum β-lactamases (ESBL)-*Enterobacterales* and are potential sources of colonization and infection (Chereau et al., 2015). The poor hygiene conditions that usually exist in hospitals in low-income countries, limited access to diagnostic tools, and reduced availability of second line antibiotics may promote the horizontal transmission of multidrug-resistant bacteria (Breurec et al., 2016; Bernabé et al., 2017). Other studies have shown this resistance is widespread on farms where animals are raised with the use of antibiotics as growth promoters (Vounba et al., 2019).

In a previous work, we collected samples from wildlife chimpanzees (*Pan troglodytes verus*) and termites in Senegal (Baron et al., 2021). We isolated enterobacteria carrying multiple antibiotic resistance genes from which we identified international high-risk clones (ST307 and ST147) of carbapenemase-producing *Klebsiella pneumoniae*. The high similarities between plasmids found in isolates from chimpanzees and termites suggest lateral gene exchanges between these two species. In this work, we pursued by exploring antibiotic resistance in other *Enterobacterales* isolated from the same specimens. We completed our study by analyzing another collection of samples isolated from Senegal wildlife [green monkeys (*Chlrocebus sabaeus*), baboons (*Papio papio*), chimpanzees (*Pan troglodytes verus*), and non-fecal environmental samples].

The objective of this work was to determine the level of resistance to antibiotics used in humans in *Enterobacterales* isolated from samples from wildlife in Senegal and to determine the genetic support of this resistance. Interestingly, we identified a transposon in *Escherichia coli* strains, located on a plasmid and in the chromosome. This observation supports the role of wildlife as a reservoir of resistance genes and as a place of exchange.

## Materials and Methods

### Sample Collection

Between 26th and 30th of August 2019, 215 non-human primates (NHPs) fecal samples (110 baboons, 95 green monkeys, and 10 chimpanzees) and 113 non-fecal environmental samples (47 termites, 42 soil samples from termite mounds, and 24 samples including fruits, other plant parts, a centipede and a maggot) were collected in four sites in Senegal. The NHPs fecal samples were collected in three sites located in southern Senegal in the Niokolo-Koba National Park: Simenti, Dar Salam, and Niokolo Poste sites. The sample collectors observed the monkeys all day to collect their fresh stool in real time so that they could be sure that it belonged to that NHPs and not to others. The non-fecal environmental samples, with the exception of the centipede and the maggot, were NHP foods, were collected in one site in south-eastern Senegal, the Dindefelo Community Nature Reserve (12°22′01.4′′N, 12°18′00.0′′W), which is located on the border with Guinea Conakry about 35 km from the town of Kedougou. The permission to collect these samples was approved by the Direction des Parcs Nationaux and Direction des Eaux, Forêts, Chasse et de la Conservation des Sols of the Senegal Ministry for the Environment and Sustainable Development (002737/DEFCCS/DGF de la Direction des Eaux, Forêts, Chasses et de la Conservation des Sols du 27/06/2019). The monkey cohort consisted of 95 green monkey samples (*Chlrocebus sabaeus*) (Linnaeus, 1766) and 110 Guinea baboon samples (*Papio papio*) (Desmarest, 1820), while the ape cohort consists of 10 chimpanzee samples (*Pan troglodytes verus*)(Schwarz, 1934) (Primates: Hominidae). It should be noted that the morphological and molecular identifications of the non-fecal environmental samples have previously been reported (Baron et al., 2021).

In addition, fecal samples collected in 2015 from Niokolo Koba National Park, Senegal, were added to this study. This cohort included seven Guinea baboon samples and four green monkey samples. Permission to collect samples was granted by the National Parks Direction and Direction des Eaux, Forêts, Chasse et de la Conservation des Sols of the Senegal Ministry for the Environment and Sustainable Development (001914/DEF/DGF de la Direction des Eaux, Forêts, Chasses et de la Conservation des Sols du 05/06/2016). No other permissions were required, as this research was non-invasive and the collection of the samples did not disrupt the wild fauna. Samples were directly stored at +4°C and cultured 2 days after collection.

### Sample Culture

Non-human primates samples were aliquoted and enriched in Tryptone Soy Broth (TSB, BioMérieux, Marcy l’Etoile, France) at 37°C for 72 h. Then, 20 μl was then inoculated on antibiotic-containing media: an LBJMR (Lucie Bardet Jean-Marc Rolain) plate (containing 4 μg/ml colistin and 100 μg/ml vancomycin) (Bardet et al., 2017), MacConkey (BioMérieux) + ertapenem (0.5 μg/ml) and MacConkey + cefotaxime (1 μg/ml). The critical concentrations selected were based on the European Committee on Antimicrobial Susceptibility Testing (EUCAST) guidelines (Version 11.0). Inoculation was followed by incubation at 37°C for 24 h. Colonies that grew on the different media were replicated on Columbia agar + 5% sheep blood (BioMérieux) for further analyses. Bacterial identification was performed by Matrix Assisted Laser Desorption Ionization-Time of Flight (MALDI-TOF) mass spectrometry (Bruker Daltonik, Bremen, Germany), as described previously (Seng et al., 2009). Concerning non-fecal environmental samples, cultures were initially inoculated on MacConkey + cefotaxime and MacConkey + ertapenem and described in a previous study (Baron et al., 2021). Briefly, samples were crushed and put in TSB at 37°C for 72 h, then 20 μl were inoculated on selective media. In this study, we also cultured the non-fecal environmental samples on LBJMR media. Unique and common *Enterobacterales* strains of NHPs and the non-fecal environmental samples were visualized by Cytoscape program to show interaction between the two ecosystems (Shannon et al., 2003).

### Antibiotic Susceptibility Test

Antibiotic Susceptibility Test (AST) were performed using the disk diffusion method following EUCAST recommendations. In case of resistance observed in disk diffusion method, minimum inhibitory concentrations (MIC) of imipenem and ertapenem were determined using the E-test method (BioMérieux), while colistin MIC was determined using the UMIC microdilution method (Biocentric, Bandol, France). The ESBL profile was detected by the observation of a champagne-cork or a keyhole between a third or fourth generation cephalosporin and clavulanic acid (Drieux et al., 2008). The ß-CARBA test (Biorad, Hercules, CA, United States) was performed to identify the strain with carbapenemase activity.

### Genomic and Bioinformatic Analysis of Genomes of Interest

The DNA of enterobacterial isolates was extracted with the BioRobot EZ1 (Qiagen, Courtaboeuf, France) using a commercial EZ1 DNA extraction kit (Qiagen) and quantified by a Qubit assay (Life Technologies, Carlsbad, CA, United States). *De novo* sequencing was performed using MiSeq technology (Illumina Inc., San Diego, CA, United States) in a paired-end strategy. Libraries were prepared using the Nextera XR DNA sample prep kit (Illumina). Briefly, DNAs were fragmented and tagged with adapters and dual-index barcodes. Then, DNAs were purified using the AMPure XP beads (Beckman Coulter, Inc., Fullerton, CA, United States) and pooled in equimolar concentrations. An Illumina-generated PhiX control libraries was added to the libraries that was then 2 × 250 bp paired-end sequenced on an Illumina Miseq. Genomes have been deposited under the Bioproject number PRJNA738374 on the NCBI Database. The three *K*. *pneumoniae* strains (Q1947, Q1948, and Q1945) described previously (Baron et al., 2021) were deposited under genome accession numbers GCA_903166445.1, GCA_903166485.1, and GCA_903166415.1, respectively. They were then assembled using Spades (Galaxy Version 3.12.0 + galaxy1) (Bankevich et al., 2012), and annotated with Prokka (Galaxy Version 1.14.6 + galaxy1) (Seemann, 2014) using the Galaxy platform.^1^ The detection of AMR genes, plasmids and virulence genes was performed with Abricate (Galaxy Version 1.0.1) using Resfinder, Plasmidfinder and VFDB databases, respectively. The sequence type (ST) of the isolates was determined using the pubMLST^2^ and Pasteur Institute databases.^3^ Pangenome analysis was performed using the Roary software (version 3.13.0) while preserving the default settings (95%: the minimum percentage of identity and 99%: a gene needs to be in to be core). The analysis included 51 genomes of sub-Saharan African strains, isolated from human, animal and the environment, that were available in PATRIC database (version 3.6.12) (accession numbers and genome details are available in Supplementary Table 1). We then used Fast tree software (version 2.1.10) to build a maximum likelihood phylogenetic tree with predefined parameters. It was computed using iTOL.^4^ A core single nucleotide polymorphism (SNP) was performed using snippy (Galaxy Version 4.6.0 + galaxy0) for the mostly related strains. We sequenced the genomes of two strains, Q2160 (ST224) and Q2170 (ST10), using a MinION sequencer (Oxford Nanopore Technologies Inc., Oxford, United Kingdom) and assembled genomes using both Illumina and MinION reads to reconstitute the IncY plasmid and compare it to the transposon of the Q2170 isolate. Bacteriophages were detected using PHASTER (Arndt et al., 2016). The reconstitution of the genetic environment was performed using the Easyfig program (Sullivan et al., 2011).

### Conjugation Experiment

This experiment was done using the Q2160 *E*. *coli* strain containing the IncY plasmid as a donor strain and an acid-resistant *E*. *coli* J53 (F- pro Azi r) as the receiver strain. The strains were enriched in TSB for 24 h then mixed in the same tube (1 ml of the donor strain in 9 ml of the receiver strain) for another 24 h. Transconjugants were selected on LB agar, supplemented by 120 μg/mL of sodium acid and 1 μg/mL of cefotaxime (Sennati et al., 2016).

## Results

### Sample Culture

**FIGURE 1:**
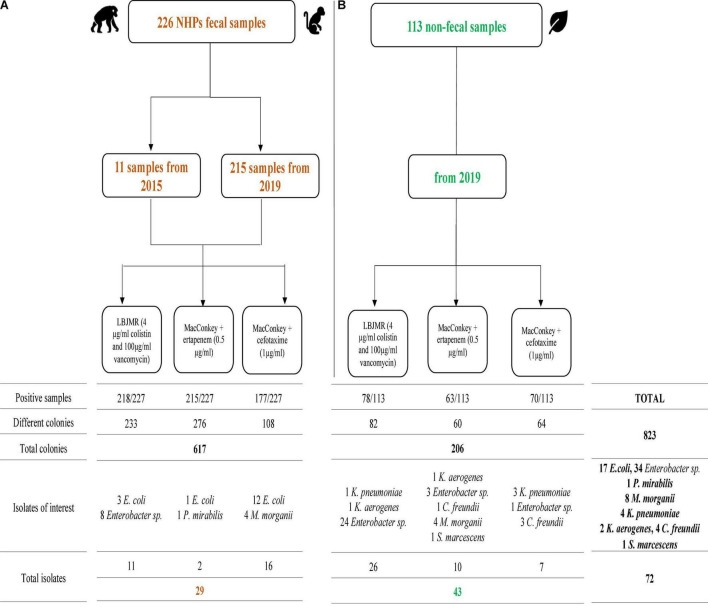
Summary of positive non-human primates (NHPs) fecal samples **(A)** and non-fecal environmental samples **(B)** cultures on different selective media. Isolates of interest are those that were sequenced.

From the 226 NHP samples, we isolated 617 bacteria including 590 Gram negative bacteria (GNB) and 27 Gram positive bacteria (GPB). The detail of the number of bacterial isolates on the different media is shown in Supplementary Table 2 and Figure 1. Among these GNB, 234 were *Enterobacterales*, of which 29 that are not naturally resistant to the antibiotics in consideration, were studied in this work. Briefly, two of these 29 *Enterobacterales* grew on MacConkey + ertapenem medium: one *E*. *coli*, and one *Proteus* sp. On MacConkey + cefotaxime media, we isolated 16 *Enterobacterales* (12 *E*. *coli* and four *Morganella morganii*). Finally, on LBJMR (Lucie Bardet Jean-Marc Rolain) medium, we isolated three *E*. *coli* and eight *Enterobacter* sp. (Three *E*. *cloacae*, three *E*. *quasiroggenkampii*, one *E*. *asburiae*, and one *E*. *bugandensis*) (Supplementary Tables 3, 4).

**FIGURE 2:**
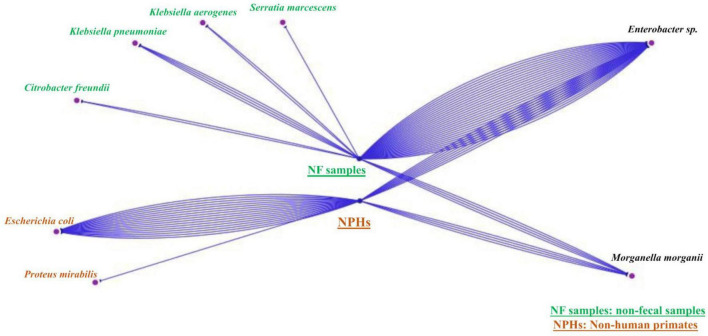
Simple view of the Cytoscape visualizing unique and common species of the two samples’ types (NHPs and non-feacal samples). The blue lines represent the number of strains found each type. There are strains that belong just to non-fecal environmental samples, others that belong just to NHPs and strains that can be found in both of them.

From the 113 non-fecal environmental samples, we isolated 206 bacteria including 201 GNB and 5 GPB. The detail of the number of bacterial isolates on the different media is shown in Supplementary Table 5 and Figure 1. Among the GNB, 84 were *Enterobacterales*, of which 43 non-naturally resistant were studied in this work. Briefly, ten *Enterobacterales* grew on MacConkey + ertapenem, including four *M*. *morganii*, three *Enterobacter sp*. (one *E*. *quasiroggenkampii*, one *E*. *cloacae*, and one *E*. *hormaechei*), one *Citrobacter cronae*, one *Klebsiella aerogenes*, and one *Serratia marcescens*. On MacConkey + cefotaxime, we isolated three *K*. *pneumoniae*, two *C*. *cronae*, and two *Enterobacter* sp. (one *E*. *quasiroggenkampii* and one *E*. *hormaechei*). Finally, on LBJMR, 25 *Enterobacter sp*. (nine *E*. *cloacae*, eight *E*. *quasiroggenkampii*, four *E*. *bugandensis*, two *E*. *roggenkampii*, and two *E*. *sichuanensis*), one *K*. *aerogenes* and one *K*. *pneumoniae* were isolated. Identical strains from the same origin are reported once. It should be noted that culture results from non-fecal environmental samples on MacConkey + ertapenem, MacConkey + cefotaxime, and MacConkey media alone have previously been described (Baron et al., 2021), but the strains of interest were sequenced for this study. Specific and common species between samples were represented in Figure 2.

### Antibiotic Susceptibility Test

A total of 72 *Enterobacterales* were isolated from NHPs and non-fecal environmental samples. We confirmed the phenotypic resistance of these isolates by antibiotic susceptibility tests (AST) (Supplementary Table 4). Sixteen isolates were confirmed to be resistant to cefotaxime (N = 16/339 samples, 5%), two resistant to ertapenem (N = 2/340 samples, 0.6%), and 37 resistant to colistin (N = 37/339 samples, 11%). The ß-carba-test was negative for the two ertapenem-resistant isolates. These antibiotic-resistant isolates were isolated from different NHP species and the environment. In chimpanzees, we isolated one ESBL-producing *E*. *coli* and six colistin-resistant *Enterobacter* sp. In green monkeys (*Chlorocebus sabaeus*), we identified seven *E*. *coli* and one *Morganella morganii* which produced ESBL, and one colistin-resistant *Enterobacter cloacae*. Four ESBL-producing and two colistin-resistant *E*. *coli* as well as one *Enterobacter asburiae* were found in Guinea baboons (*Papio papio*). From the non-fecal environmental samples, we isolated two ertapenem-resistant *M*. *morganii*, *27* colistin-resistant *Enterobacterales* including 25 *Enterobacter* sp., one *K*. *pneumoniae* and one *K*. *aerogenes* and three ESBL-producing *K*. *pneumoniae*.

Of these 72 *Enterobacterales*, 23 had a multidrug resistant profile (MDR) (Magiorakos et al., 2012) and were resistant to four or more antibiotic families, 46 to fewer than four antibiotic families, and three had no resistance. Resistance to third generation cephalosporins (3GC) was 29% (N = 21/72) whereas colistin resistance represented 65% (47/72) of the total strains. 3GC-resistant isolates, had co-resistance with tetracycline family (52%, N = 11/21) (doxycycline), fluoroquinolone (43%, N = 9) (ciprofloxacin) and trimethoprim-sulfamethoxazole (62%, N = 13). Resistance to amoxicillin was found in 75% of the *E*. *coli* strains (N = 12/16).

### Population Analysis of Bacterial Species

We sequenced the genome of the 72 *Enterobacterales* and studied clonal population and AMR genes. Details of the assembly results are provided in Supplementary Table 4. The population distribution of *Enterobacterales* was mainly represented by new sequence types (STs), indicating the existence of a population specific to the ecosystem analyzed but we found clonal complexes that are associated with human infections for *E*. *coli* and *K*. *pneumoniae*. Of the 16 *E*. *coli*, six belonged to ST10, two to ST224, and nine to other unique STs (ST212, ST202, ST196, ST469, ST2803, ST642, ST10648, ST3580, and ST6611). For *K*. *pneumoniae*, three belonged to ST307 and had previously been described (Baron et al., 2021), but one had a new ST5460. Of the 37 *Enterobacter sp*. sequenced, only three strains belonged to a known ST (ST1084- Q3805, ST113- Q2141, and ST565-Q2153), while the remaining 34 isolates belonged to 29 new different STs. Interestingly, one group was containing strains belonged to the chimpanzee and non-fecal environmental samples. It consisted of 11 STs (ST1545, ST1547, ST1548, ST1549, ST1552, ST1554, ST1563, ST1565, ST1566, ST1567, and ST1570) combining strains of the environment origin (N = 7) and strains from chimpanzees (N = 4). Finally, two *K*. *aerogenes* and two *C*. *cronae* from the non-fecal environmental samples belonged to new STs ST225 and ST226 for *K*. *aerogenes* and ST574 and ST575 for *C*. *cronae* (Supplementary Table 4).

### Antimicrobial Resistant Gene Circulation in Antibiotic-Resistant Isolates

**FIGURE 3:**
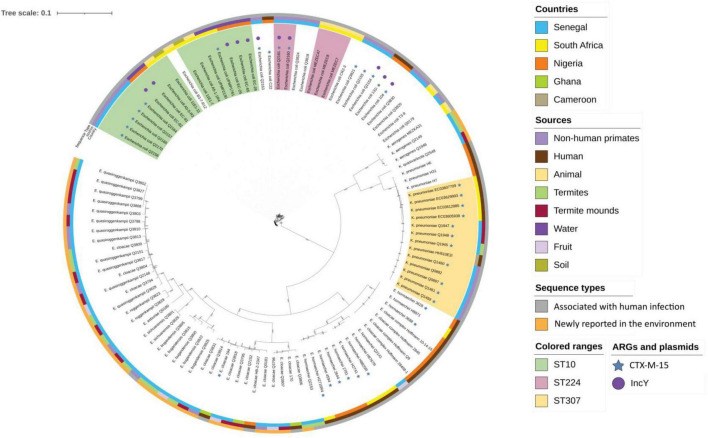
Maximum likelihood phylogenetic tree representation of the 9 *Klebsiella* spp., 16 *E*. *coli*, and 37 *Enterobacter* spp. strains compared with strains from different origin of the Sub-Saharan region. The tree was generated and annotated with the iTOL tool (https://itol.embl.de/tree/).

**FIGURE 4:**
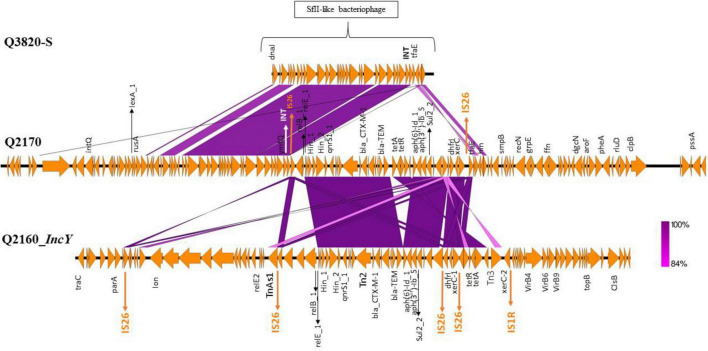
Genomic comparison of the SFII-like bacteriophage region of the 3GC-susceptible *Escherichia coli* strain (Q3820), the transposon integrated in the SFII-like prophage region of the ST10 3GC-resistant *E*. *coli*(Q2170) and the IncY plasmid sequence harboring the same transposon of the ST224 *E*. *coli* (Q2160). This comparison shows the putative transfer of resistance genes between these two strains. The figure was made using the Easyfig program.

We noticed that 53 of the 72 *Enterobacterales* had four or more resistance genes, some had the same association of genes, such as *E*. *coli* and *K*. *pneumoniae* (Supplementary Table 4 and Table 1). We found a CTX-M-15 type β-lactamases in 12 *E*. *coli* from NHPs, three ST307 *K*. *pneumoniae* isolated from the environment (Figure 3). Four of the 12 *E*. *coli* (2 ST224, 1 ST202, and ST469) harbored an *IncY* plasmid of 91,959 bp length (GC% = 52.06%) carrying resistance genes to beta-lactam (*bla*_*CTX*–*M*–15_, *bla*_*TEM*1*B*_), sulfonamide (*sul2*, *dfrA14*), fluroquinolones (*qnrs*), tetracycline (*tetA*, *tetR*) and aminosides [*aph*(*3′′*), *aph*(*6*)]. The plasmid IncY was non conjugative, did not have a complete conjugative apparatus, and conjugation to an *E*. *coli* J53 failed on three attempts. It was found in three green monkey and one baboon samples (Figure 3). A Tn2 transposon of 30,340 bp (GC% = 52.05%) containing the same AMR genes found on the IncY plasmid [*bla*_*CTX*–*M*–15_, *bla*_*TEM*1*B*_, *sul2*, *dfrA14*, *qnrs1*, *aph*(*3′′*), *aph*(*6*), *tetA*, and *tetR*] was observed in the chromosome of four of the six ST10 isolates (Q2156, Q2170, Q2165, and Q2157). These four strains differed from 1 to 14 SNPs in their core genome suggesting they had the same origin (Figure 3). Interestingly, this transposon was similar to that present in the plasmid IncY (Figure 4) but was inserted in a SfII-like prophage (KC736978) of 35.5 bp (GC% = 50.23%) in the chromosome of the ST10 isolate (Q2170). On the 5′–3′ locus, an insertion sequence (IS)26 inserted in an integrase (INT) of the phage, causing a deletion of 8 bp in the integrase sequence, shifting the reading frame and leading to the synthesis of 187 amino acid residues of the integrase protein against 216 amino acid residues in the reference phage. On the 3′–5′ locus, the IS26 inserted before the tail fiber assembly protein (TfaE), leading to the truncation of a hypothetical protein located between the integrase (INT) and the TfaE protein. Interestingly, the intact SFII-like prophage was found in a β-lactamases susceptible *E*. *coli* isolate (Q3820) that was only resistant to colistin (Figure 4). Finally, the four other *E*. *coli* isolates carrying CTX-M-15 enzymes belonged to different STs [two ST10 (Q2157, Q2164), one ST3580 (Q2155), and one ST212 (Q3821)], had no IncY plasmid and did not have the same transposon. Three of them carried one to five plasmids (IncF, Col, and IncB plasmids, details in Supplementary Table 4), but the location of *bla*_*CTX–M–*15_ on the plasmids could not be identified in these genomes. The conjugation experiment showed negative results as no growth has been detected for a J53 *E*. *coli* receiving the cefotaxime resistant gene.

Regarding other *Enterobacterales*, three *C*. *cronae* genomes harbored a fluroquinolone-resistance gene (*qnrB12*) as well as the chromosomal beta-lactamase *bla*_*CMY* –98_1._ In the 37 *Enterobacter* spp., we identified four different chromosomal *ampC* genes, namely *bla*_*CMH–3–1*_ (N = 10), *bla*_*ACT–*6–1_ (N = 10), *bla*_*CMG–1*_ (N = 14), and *bla*_*MIR–1*_ (N = 2). None of these strains had an inducible expression of their AmpC β-lactamase with a phenotypic resistance to 3 GC. In the eight *M*. *morganii* genomes isolated from green monkeys and non-fecal environmental samples, we found the *ampC* β-lactamase (*bla*_*MOR–2*_, *bla*_*DHA12*_), conferring resistance to 3 GC. The summary of antibiotic resistance genes in these strains is presented in Table 1. The *bla*_*CMG*_ gene shows 98.34% similarity with *bla*_*ACT–62*_ suggesting that they could be allelic variants of the same AmpC gene.

**TABLE 1:** Summary of antibiotic resistance genes in isolates of interest.

Species	Strain number	Samples	Sample origin	MLST	Antibiotic resistance genes
*Escherichia coli*	Q2154	SV 002	Green monkeys	ST469	*mdf*(*A*), *sul2*, *aph* (*3′′*)*-Ib*, *aph*(*6*)*-Id*, *blaTEM-1B*, *tet*(*A*), *dfrA14*, *blaCTX-M-15*, and *qnrS1*
*Escherichia coli*	Q2155	SV 003	Green monkeys	ST3580	*aac*(*6*′)*-Ib-cr*, *blaCTX-M-15*, *qnrS1*, *mdf*(*A*), *dfrA17*, *aadA5*, and *mph*(*A*)
*Escherichia coli*	Q2156	SV 005	Green monkeys	ST10	*qnrS1*, *blaCTX-M-15*, *blaTEM-1B*, *mdf*(*A*), *sul2*, *aph*(*3′′*)*-Ib*, *aph*(*6*)*-Id*, *tet*(*A*), and *dfrA14*
*Escherichia coli*	Q2157	SV 006	Green monkeys	ST10	*tet*(*A*), *dfrA14*, *mdf*(*A*), *blaTEM-1B*, *blaCTX-M-15*, *qnrS1*, *aph*(*6*)*-Id*, *aph*(*3′′*)*-Ib*, and *sul2*
*Escherichia coli*	Q2160	SV 054	Green monkeys	ST224	*mdf*(*A*), *tet*(*A*), *sul2*, *aph*(*3′′*)*-Ib*, *aph*(*6*)*-Id*, *blaTEM-1B*, *blaCTX-M-15*, *qnrS1*, and *dfrA14*
*Escherichia coli*	Q2170	SV 066	Green monkeys	ST10	*mdf*(*A*), *sul2*, *aph*(*3′′*)*-Ib*, *aph*(*6*)*-Id*, *tet*(*A*), *blaTEM-1B*, *blaCTX-M-15*, *qnrS1*, and *dfrA14*
*Escherichia coli*	Q2161	SV 069	Green monkeys	ST224	*mdf*(*A*), *sul2*, *aph*(*3′′*)*-Ib*, *aph*(*6*)*-Id*, *blaTEM-1B*, *blaCTX-M-15*, *qnrS1*, *tet*(*A*), and *dfrA14*
*Escherichia coli*	Q0179	MFB 3	Baboons	ST2803	*mdf*(*A*)
*Escherichia coli*	Q2163	BG 007	Baboons	ST202	*mdf*(*A*), *qnrS1*, *blaCTX-M-15*, *blaTEM-1B*, *aph*(*6*)*-Id*, *aph*(*3′′*)*-Ib*, *sul2*, *tet*(*A*), and *dfrA14*
*Escherichia coli*	Q3820	BG 034	Baboons	ST6611	*ant*(*3′′*)*-Ia*, *tet*(*A*), and *mdf*(*A*)
*Escherichia coli*	Q3819	BG 046	Baboons	ST196	*mdf*(*A*)
*Escherichia coli*	Q2164	BG 049	Baboons	ST10	*mdf*(*A*), *tet*(*A*), *qnrS1*, and *blaCTX-M-15*
*Escherichia coli*	Q3830	BG 082	Baboons	ST10648	*mdf*(*A*)
*Escherichia coli*	Q2165	BG 085-A	Baboons	ST10	*mdf*(*A*), *sul2*, *aph*(*3′′*)*-Ib*, *aph*(*6*)*-Id*, *tet*(*A*), *blaTEM-1B*, *blaCTX-M-15*, *qnrS1*, and *dfrA14*
*Escherichia coli*	Q3824	BG 104	Baboons	ST642	*mdf*(*A*)
*Escherichia coli*	Q3821	CH 002	Chimpanzees	ST202	*qnrS1*, *blaCTX-M-15*, and *mdf*(*A*)
*Morganella morganii*	Q2158	SV 017	Green monkeys	–	*blaMOR-2*, *catA2*, and *tet*(*D*)
*Morganella morganii*	Q2159	SV 028	Green monkeys	–	*blaMOR-2*
*Morganella morganii*	Q2169	SV 054	Green monkeys	–	*catA2* and *blaDHA-12*
*Morganella morganii*	Q2162	SV 094	Green monkeys	–	*blaDHA-12*, *tet*(*D*), and *catA2*
*Morganella morganii*	Q2142	NCS 002B	Plant	–	*blaDHA-12* and *catA2*
*Morganella morganii*	Q2143	NCS 002C	Plant	–	*catA2*, *blaDHA-12*
*Morganella morganii*	Q2144	NCS 003A	Plant	–	*blaDHA-12*, *catA2*
*Morganella morganii*	Q2147	NCS 010	Plant	–	*blaDHA-12* and *catA2*
*Proteus mirabilis*	Q3812	SV 035	Green monkeys	–	*tet*(*J*) and *cat*
*Serratia ureilytica*	Q2145	NCS 006A	Termites	–	*aac*(*6*′)*-Ic*, *blaSST-1*, *oqxB*, and *tet*(*41*)
*Citrobacter cronae*	Q2146	NCS 009	Fruit	ST574	*qnrB12* and *blaCMY-98*
*Citrobacter cronae*	Q2150	NCS 033	Termites	ST575	*qnrB12* and *blaCMY-98*
*Citrobacter cronae*	Q2168	NCS 037 (1)	Termites	ST575	*qnrB34* and *blaCMY-98*
*Enterobacter asburiae*	Q0180	MFB 6	Baboons	ST1543	*fosA*, *oqxB*, *oqxA*, and *blaACT-6*
*Enterobacter cloacae*	Q0181	AGM 2	Green monkeys	ST1544	*fosA*, *oqxB*, *oqxA*, and *blaCMH-3*
*Enterobacter quasiroggenkampii*	Q3813	CH 001	Chimpanzees	ST1545	*oqxA*, *oqxB*, *fosA*, and *blaCMG*^1^
*Enterobacter cloacae*	Q3814	CH 002	Chimpanzees	ST1546	*oqxA*, *oqxB*, *fosA*, and *blaCMH-3*
*Enterobacter quasiroggenkampii*	Q3810	CH 003	Chimpanzees	ST1547	*oqxA*, *oqxB*, *fosA*, and *blaCMG*^1^
*Enterobacter cloacae*	Q3804	CH 005	Chimpanzees	ST1548	*oqxA*, *oqxB*, *fosA*, and *blaCMG*^1^
*Enterobacter bugandensis*	Q3805	CH 009	Chimpanzees	ST1084	*oqxB*, *oqxA*, *fosA*, and *blaACT-6*
*Enterobacter quasiroggenkampii*	Q3803	CH 010	Chimpanzees	ST1549	*oqxA*, *oqxB*, *fosA*, and *blaCMG*^1^
*Enterobacter hormaechei*	Q2141	NCS 001	Fruit	ST113	*blaTEM-1B*, *aph*(*6*)*-Id*, *aph*(*3′′*)*-Ib*, *oqxB*, *oqxA*, *fosA*, *blaACT-15*, *sul2*, *dfrA14*, and *tet*(*D*)
*Enterobacter bugandensis*	Q3822	NCS 005	Fruit	ST1550	*blaACT-6*, *oqxA*, *oqxB*, and *fosA*
*Enterobacter cloacae*	Q3807	NCS 006B	Termite mounds	ST1551	*oqxA*, *oqxB*, *fosA*, and *blaCMH-3*
*Enterobacter quasiroggenkampii*	Q2148	NCS 013B	Termite mounds	ST1552	*oqxA*, *oqxB*, *fosA*, and *blaCMG*^1^
*Enterobacter cloacae*	Q3806	NCS 018B	Termites	ST1553	*oqxA*, *oqxB*, *fosA*, and *blaCMH-3*
*Enterobacter quasiroggenkampii*	Q3808	NCS 019B	Termite mounds	ST1554	*oqxA*, *oqxB*, *fosA*, and *blaCMG*^1^
*Enterobacter cloacae*	Q3794	NCS 022A	Fruit	ST1570	*oqxA*, *oqxB*, *fosA*, and *blaCMG*^1^
*Enterobacter cloacae*	Q3796	NCS 023	Fruit	ST1555	*oqxA*, *oqxB*, *fosA*, and *blaCMH-3*
*Enterobacter cloacae*	Q3818	NCS 24A	Fruit	ST1556	*oqxA*, *oqxB*, *fosA*, and *blaCMH-3*
*Enterobacter cloacae*	Q3831	NCS 027C	Termite mounds	ST1557	*oqxA*, *oqxB*, *fosA*, and *blaCMH-3*
*Enterobacter sichuanensis*	Q3826	NCS 028B	Termites	ST1558	*oqxA*, *oqxB*, *fosA*, and *blaACT-6*
*Enterobacter roggenkampii*	Q3823	NCS 030C	Termite mounds	ST1559	*oqxA*, *oqxB*, *fosA*, and *blaMIR-1*
*Enterobacter cloacae*	Q3809	NCS 031A	Termites	ST1565	*fosA*, *blaCMG*^1^, *oqxA*, and *oqxB*
*Enterobacter quasiroggenkampii*	Q3802	NCS 034A	Termites	ST1554	*oqxA*, *oqxB*, *fosA*, and *blaCMG*^1^
*Enterobacter quasiroggenkampii*	Q3827	NCS 034B	Termite mounds	ST1554	*oqxA*, *oqxB*, *fosA*, and *blaCMG*^1^
*Enterobacter bugandensis*	Q3815	NCS 035A	Termites	ST1561	*oqxA*, *oqxB*, *fosA*, and *blaACT-6*
*Enterobacter sichuanensis*	Q3801	NCS 036A	Termite mounds	ST1562	*oqxA*, *oqxB*, *fosA*, and *blaACT-6*
*Enterobacter quasiroggenkampii*	Q2151	NCS 037A	Termites	ST1563	*oqxA*, *oqxB*, *fosA*, and *blaACT-6*
*Enterobacter cloacae*	Q2152	NCS 039B	Termite mounds	ST1564	*oqxA*, *oqxB*, *fosA*, and *blaCMH-3*
*Enterobacter quasiroggenkampii*	Q3799	NCS 040 B	Termite mounds	ST1554	*oqxA*, *oqxB*, *fosA*, and *blaCMG*^1^
*Enterobacter quasiroggenkampii*	Q3798	NCS 045C	Termite mounds	ST1566	*oqxA*, *oqxB*, *fosA*, and *blaCMG*^1^
*Enterobacter quasiroggenkampii*	Q3817	NCS 046B	Termite mounds	ST1567	*oqxA*, *oqxB*, *fosA*, and *blaCMG*^1^
*Enterobacter bugandensis*	Q3825	NCS 049A	Termites	ST1568	*oqxA*, *oqxB*, *fosA*, and *blaACT-6*
*Enterobacter cloacae*	Q3795	NCS 050D	Termite mounds	ST1569	*oqxA*, *oqxB*, *fosA*, and *blaCMH-3*
*Enterobacter hormaechei*	Q2153	NCS 050 (19)	Termites	ST565	*oqxA*, *oqxB*, *fosA*, and *blaACT-16*
*Enterobacter quasiroggenkampii*	Q3828	NCS 051A	Termites	ST1570	*oqxA*, *oqxB*, *fosA*, and *blaCMG*^1^
*Enterobacter roggenkampii*	Q3829	NCS 053A	Termites	ST1571	*oqxA*, *oqxB*, *fosA*, and *blaMIR-2*
*Enterobacter bugandensis*	Q3800	NCS 053B	Termites	ST1572	*oqxA*, *oqxB*, *fosA*, and *blaACT-6*
*Klebsiella aerogenes*	Q1946	NCS 028A	Termites	ST225	*oqxA*, *oqxB*, and *fosA*
*Klebsiella aerogenes*	Q2149	NCS 033A	Termites	ST226	*fosA*, *oqxA*, and *oqxB*
*Klebsiella quasivariicola*	Q2548	NCS 42B	Termite mounds	ST5460	*oqxA*, *oqxB*, *fosA*, and *blaLEN26*
*Klebsiella pneumoniae*	Q1947	NCS 043A	Termite mounds	ST307	*blaTEM-1B*, *blaSHV-106*, *blaOXA-1*, *blaCTX-M-15*, *aac*(*3*)*-IIa*, *aac*(*6*′)*Ib-cr*, *aac*(*6*′)*Ib-cr*, *qnrB1*, *dfrA14*, *sul2*, and *tet*(*A*)
*Klebsiella pneumoniae*	Q1948	NCS 043B	Termite mounds	ST307	*blaTEM-1B*, *blaSHV-106*, *blaOXA-1*, *blaCTX-M-15*, *aac*(*3*)*-Iia*, *aac*(*6*′)*Ib-cr*, *aac*(*6*′)*Ib-cr*, *qnrB1*, *dfrA14*, *sul2*, and *tet*(*A*)
*Klebsiella pneumoniae*	Q1945	NCS 045A	Termites	ST307	*blaTEM-1B*, *blaSHV-106*, *blaOXA-1*, *blaCTX-M-15*, *aac*(*3*)*-Iia*, *aac*(*6*′)*Ib-cr*, *aac*(*6*′)*Ib-cr*, *qnrB1*, *dfrA14*, *sul2*, and *tet*(*A*)

*^1^These sequences have 99.62% identity with bla_CMG_ (accession number AY266892) and 98.34% with bla_ACT–62_ (MH469270.1).*

### Genome Comparison With Published Bacterial Genomes From Sub-Saharan Africa From Human, Animal, and Environmental Origins

We compared the genomes of *E*. *coli*, *K*. *pneumonia*, and *Enterobacter* spp. isolated in our work with 51 genomes from different origin isolated in sub-Saharan Africa (Figure 3). *Enterobacter* isolates clustered together, far from the human isolates which were consistent with the ST results. *E*. *coli* and *K*. *pneumoniae* isolates were more related with human isolates but their core genome differed by more than 20,000 SNPs suggesting there were not directly related. We have also included in this analysis two strains of *E*. *coli* (131i and 104) that were isolated from animals use for human consumption in Nigeria (Sharma et al., 2020) that were interesting because they carried an IncY plasmid, the antibiotic resistance genes included in the transposon [*bla*_*CTX*–*M*–15_, *bla*_*TEM*1*B*_, *sul2*, *dfrA14*, *qnrs1*, *aph*(*3′′*), *aph*(*6*), *tetA* and *tetR*] and a SfII-like prophage. However, these isolates did not cluster with isolates that contained the transposon (Q2154, Q2156, Q2160, Q2161, Q2163, Q2165, Q2157, and Q2170). Unfortunately, we were not able to find the antibiotic resistance genes location in these two isolates.

## Discussion

Microbial diversity in natural environments is considered to be the largest unexplored reservoir of biodiversity on earth. Many studies have described bacterial diversity in primates, but few of them have focused on their genomic characteristics. This explains the large number of new STs of *Enterobacter* sp., *C*. *cronae*, and *K*. *aerogenes* found in our study. *E*. *coli* [ST10 (Mohsin et al., 2017) and ST224 (Cao et al., 2014)] and *K*. *pneumoniae* [ST307 and ST147 (Peirano et al., 2020)] found in our previous study (Baron et al., 2021) are reported to cause human infections. Although our newly described STs have not yet been found in humans and transmission cannot be excluded.

Interestingly, in our study, 11 green monkeys, three baboons and one chimpanzee carried an *Enterobacterales* with a MDR profile, often an ESBL profile. This MDR profile was also found in the *Enterobacterales* of nine non-fecal environmental samples showing resistance to 3GC, tetracycline and fluoroquinolones. Moreover, the rate of resistance was relatively high for a cohort far from massive antibiotic exposure, especially for colistin but also for 3GC (29%, N = 21/72). Moreover, 18% (N = 13/72) of the isolated strains were resistant to tetracycline and 18% (N = 13/72) were resistant to sulphonamides. According to the WHO report published in 2014 highlighting resistance levels to the most widely-used antibiotics, fluoroquinolones and 3GC antibiotics are known to be less used in Africa, where the resistance rate is lower than in Europe (Bassoum et al., 2019). Our results show that 13% (N = 9/72) of the isolates were fluoroquinolone-resistant and 29% (21/72) were C3G-resistant. These phenotypic results were confirmed genotypically, where AMR genes were found to be consistent with isolate antibiotic resistance. 3GC-resistance is often co-presented in this study with tetracycline, fluoroquinolone and sulphonamide resistance, conferring a MDR profile for the strain, which was demonstrated in the IncY plasmid that carries all the AMR genes conferring resistance to these antibiotics at the same time. In contrast, mobile colistin resistance (*mcr*) genes were not found and further analysis will be needed in order to decipher the resistance mechanisms of *Enterobacter* sp.

The IncY plasmid has been found in the environment (Ma et al., 2021), including in water samples (Moremi et al., 2016) and in animals such as camels in Tunisia (Saidani et al., 2019). Although it could be mobilizable *in silico* because of the current flanking sequence surrounding this DNA element, the conjugation experimental attempt to mobilize it has failed. This plasmid is often associated with ESBL-resistance genes, especially *bla*_*CTX*–*M*–15_ (Rasheed et al., 2020). Here, AMR genes clustered in this plasmid within a transposon [*bla*_*CTX*–*M*–15_, *bla*_*TEM*1*B*_, *sul2*, *dfrA14*, *qnrs1_1*, *aph*(*3′′*), *aph*(*6*), *tetA*, and *tetR*] in four *E*. *coli* strains, and were surrounded by IS26 from each side. Interestingly, we showed that this transposon was inserted in a SfII-like phage on the chromosome of a ST10 isolate (Figure 4) leading to nucleotides deletion that modified the prophage integrase (INT) amino-acid sequence from a side and truncated a hypothetical protein from the other side. Phages are believed to play a critical role in the horizontal transfer of bacterial genes, including antibiotic resistance genes (Kondo et al., 2021). The *SfII* phage is known to play a role in virulence spread (Mavris et al., 1997). This finding demonstrates that intermediate steps between transposons and the genomes of the phage can also be found and the recombination site of a prophage is vulnerable and can receive mobile elements (Brown-Jaque et al., 2015). Some studies have shown the integration of the transposon next to the recombination site of a phage (Huang et al., 2017) but none has shown the insertion of the transposon inside the phage to our knowledge. We were interested in the two *E*. *coli* strains that were isolated from poultry in Nigeria (Sharma et al., 2020) and contained the same phage, plasmid and antibiotic resistance genes that our *E*. *coli* isolates but we were unable to verify if they have the same transposon and its location. These findings, however, suggest a circulation of these mobile elements in bacteria from animals in Africa.

Antimicrobial resistant in humans is a current issue linked to the overuse of antibiotics in clinical, veterinary and agricultural practices, which has been considered to be the main selective pressure for AMR strains and AMR gene dissemination since the 1950s (Thaller et al., 2010). However, the literature is rich in cases reporting the emergence of AMR in the absence of antibiotic use, as is the case for colistin resistance (Olaitan et al., 2016). Other factors, such as anthropological and socioeconomic factors (Collignon et al., 2018) and cross-resistance with other antibiotics may be involved (Napier et al., 2013). The “One Health” approach has emerged several years ago (Thamlikitkul et al., 2015), and puts the issue of AMR in a more global context. This approach has already been well studied for farm animals but remains poorly studied for wild animals (Diallo et al., 2020). The study of NHPs is even more interesting since they are phylogenetically close to humans and may be colonized or infected by species that are close or identical to those that are pathogenic to humans (Wolfe et al., 1998). Since NHPs live in areas with different protection status and human impact, they are a good model for studying the phenomenon of AMR, especially since they also live in groups and display behaviors similar to our own.

## Conclusion

Our study shows that AMR can be found in wildlife and that this emergence, in places where human selection and pressure is reduced, is a public health concern. The wild environment is not well studied and hosts a large, undescribed microbial diversity. Transfer of several AMR genes via transposon in plasmids and via integrated prophages should be further explored to understand this dynamic in natural environments.

## Data Availability Statement

The datasets presented in this study can be found in online repositories. The names of the repository/repositories and accession number(s) can be found in the article/Supplementary Material.

## Ethics Statement

The animal study was reviewed and approved by the Direction des Parcs Nationaux and Direction des Eaux, Forêts, Chasse et de la Conservation des Sols of the Senegal Ministry for the Environment and Sustainable Development.

## Author Contributions

SB, J-MR, and DR designed the study, drafted, and revised the manuscript. OM, BD, AB, GD, and CS collected the samples. RA drafted the manuscript. RA, EK, LH, and AI performed microbiology analyses. RH-A facilitated sample collection and revised the manuscript. All authors have read and approved the final manuscript.

## Conflict of Interest

The authors declare that the research was conducted in the absence of any commercial or financial relationships that could be construed as a potential conflict of interest.

## Publisher’s Note

All claims expressed in this article are solely those of the authors and do not necessarily represent those of their affiliated organizations, or those of the publisher, the editors and the reviewers. Any product that may be evaluated in this article, or claim that may be made by its manufacturer, is not guaranteed or endorsed by the publisher.

## Footnotes

1
https://usegalaxy.org.au/

2
https://pubmlst.org/

3
https://bigsdb.web.pasteur.fr/

4
https://itol.embl.de/itol.cgi

## Supplementary Material

Supplementary Tables 1 List of reference bacterial genomes used for phylogenetic comparison (Figure 3).

Supplementary Table 2 List of bacterial isolates in NHPs fecal samples on different media.

Supplementary Table 3 Summary of the *Enterobacterales* isolated on selective media (Source, date and localisation were presented).

Supplementary Table 4 Characteristics summary of *Enterobacterales* isolated on selective media. Antibiotic susceptibility tests to provide resistance profile and genomic analysis results (antibiotic resistance genes, plasmid and virulence genes as well as allelic profile) were presented.

Supplementary Table 5 List of bacterial isolates contained in non-fecal environmental samples on different media.
