# Supplementary material for: Population Diversity of Antibiotic Resistant Enterobacterales in Samples From Wildlife Origin in Senegal: Identification of a Multidrug Resistance Transposon Carrying blaCTX–M–15 in Escherichia coli
Source: Front Microbiol. 2022 Mar 18;13:838392. doi: 10.3389/fmicb.2022.838392 (PMC8971907; doi:10.3389/fmicb.2022.838392)
Supplement: Supplementary Table 2 — List of bacterial isolates in NHPs fecal samples on different media. [file Table_2.DOCX]

**Supplementary data**

Table S2: List of bacteria grown in NHPs fecal samples on different media.

|  |  | |  |  |  |
| --- | --- | --- | --- | --- | --- |
|  | MC + ertapenem | MC + cefotaxime | | LBJMR | TOTAL |
| **GPB** | **14** | **0** | | **13** | **27** |
| *Pediococcus pentaceus* | 0 | 0 | | 4 | 4 |
| *Weissela paramesenteroides* | 0 | 0 | | 7 | 7 |
| *Lactobacillus plantarum* | 0 | 0 | | 1 | 1 |
| *Lactobacillus brevis* | 0 | 0 | | 1 | 1 |
| *Bacillus magaterium* | 1 | 0 | | 0 | 1 |
| *Enterococcus gallinarum* | 0 | 0 | | 0 | 0 |
| *Enterococcus hirae* | 0 | 0 | | 0 | 0 |
| *Enterococcus avium* | 10 | 0 | | 0 | 10 |
| *Enterococcus faecium* | 3 | 0 | | 0 | 3 |
| **GNB** | **262** | **108** | | **220** | **590** |
| *Pseudomonas monteilii* | 11 | 0 | | 0 | 11 |
| *Pseudomonas guariconensis* | 15 | 0 | | 0 | 15 |
| *Pseudomonas citronellolis* | 1 | 1 | | 0 | 2 |
| *Pseudomonas aeruginosa* | 45 | 15 | | 0 | 60 |
| *Pseudomonas nitroreducens* | 8 | 30 | | 0 | 38 |
| *Pseudomonas putida* | 24 | 0 | | 0 | 24 |
| *Pseudomonas oryzihabitans* | 1 | 0 | | 0 | 1 |
| *Stenotrophomonas maltophilia* | 56 | 2 | | 0 | 58 |
| *Acinetobacter courvalini* | 6 | 0 | | 0 | 6 |
| *Acinetobacter bereziniae* | 22 | 3 | | 0 | 25 |
| *Acinetobacter guillouie* | 7 | 0 | | 0 | 7 |
| *Acinetobacter pittii* | 31 | 3 | | 0 | 34 |
| *Acinetobacter gyllenbergi* | 4 | 0 | | 0 | 4 |
| *Acinetobacter tandoii* | 1 | 0 | | 0 | 1 |
| *Acinetobacter johsonii* | 1 | 0 | | 0 | 1 |
| *Acinetobacter nosocomialis* | 4 | 0 | | 0 | 4 |
| *Acinetobacter ursingii* | 1 | 0 | | 0 | 1 |
| *Acinetobacter gerneri* | 11 | 6 | | 0 | 17 |
| *Acinetobacter otitidis* | 1 | 0 | | 0 | 1 |
| *Acinetobacter baumannii* | 10 | 1 | | 0 | 11 |
| *Brucella intermedium* | 1 | 14 | | 2 | 17 |
| *Bordetella trematum* | 0 | 7 | | 0 | 7 |
| *Castenellaniella defragans* | 0 | 1 | | 0 | 1 |
| *Achromobacter insolitus* | 0 | 7 | | 0 | 7 |
| *Ahcromobacter xylosoxidans* | 0 | 1 | | 0 | 1 |
| ***Enterobacterales*** | **2** | **17** | | **215** | **234** |
| *Serratia marcescens* | 0 | 0 | | 6 | 6 |
| *Escherichia coli* | 1 | 13 | | 3 | 17 |
| *Enterobacter sp.* | 0 | 0 | | 8 | 8 |
| *Morganella morganii* | 0 | 4 | | 95 | 99 |
| *Proteus mirabilis* | 1 | 0 | | 56 | 57 |
| *Providencia alcalifaciens* | 0 | 0 | | 37 | 37 |
| *Providencia rettgeri* | 0 | 0 | | 10 | 10 |
| **TOTAL** | **276** | **108** | | **233** | **617** |
| **No bacterial growth** | **12** | **46** | | **13** | **71** |

MC: MacConkey
