# Supplementary material for: Population Diversity of Antibiotic Resistant Enterobacterales in Samples From Wildlife Origin in Senegal: Identification of a Multidrug Resistance Transposon Carrying blaCTX–M–15 in Escherichia coli
Source: Front Microbiol. 2022 Mar 18;13:838392. doi: 10.3389/fmicb.2022.838392 (PMC8971907; doi:10.3389/fmicb.2022.838392)
Supplement: Supplementary Table 5 — List of bacterial isolates contained in non-fecal environmental samples on different media. [file Table_5.DOCX]

**Supplementary data**

Table S5: List of bacteria grown and isolated in non-fecal samples samples on different media.

|  | Non-fecal samples | | |  |
| --- | --- | --- | --- | --- |
|  | MC + ertapenem | MC + cefotaxime | LBJMR | Total |
| **GPB** | **0** | **0** | **5** | **5** |
| *Lactobacillus plantarum* | 0 | 0 | 5 | 5 |
| **GNB** | **60** | **64** | **77** | **201** |
| *Pseudomonas monteilii* | 1 | 5 | 0 | 6 |
| *Pseudomonas aeruginosa* | 8 | 9 | 1 | 18 |
| *Pseudomonas guariconensis* | 1 | 1 | 0 | 2 |
| *Pseudomonas nitroreducens* | 2 | 3 | 0 | 5 |
| *Pseudomonas putida* | 0 | 2 | 0 | 2 |
| *Pseudomonas citronellolis* | 1 | 0 | 0 | 1 |
| *Pseudomonas orizyhabitans* | 1 | 0 | 0 | 1 |
| *Pseudomonas mosselii* | 2 | 0 | 0 | 2 |
| *Stenotrophomonas maltophilia* | 9 | 6 | 5 | 20 |
| *Acinetobacter baumannii* | 15 | 12 | 0 | 27 |
| *Acinetobacter nosocomialis* | 3 | 0 | 0 | 3 |
| *Acinetobacter pittii* | 0 | 6 | 0 | 6 |
| *Acinetobacter bereziniae* | 4 | 0 | 0 | 4 |
| *Brucella intermedium* | 4 | 4 | 0 | 8 |
| *Ochrobactrum tritici* | 0 | 1 | 3 | 4 |
| *Burkholderia cepacia* | 0 | 0 | 1 | 1 |
| *Burkholderia ambifaria* | 0 | 2 | 0 | 2 |
| *Burkholderia cenocepacia* | 1 | 0 | 0 | 1 |
| ***Enterobacterales*** | **10** | **7** | **67** | **84** |
| *Klebsiella pneumoniae* | 0 | 3 | 1 | 4 |
| *Klebsiella aerogenes* | 1 | 0 | 1 | 2 |
| *Enterobacter sp.* | 3 | 1 | 25 | 29 |
| *Citrobacter freundii* | 1 | 3 | 0 | 4 |
| *Morganella morganii* | 4 | 0 | 9 | 13 |
| *Providencia alcalifaciens* | 0 | 0 | 4 | 4 |
| *Providencia rettgeri* | 0 | 0 | 4 | 4 |
| *Serratia marcescens* | 1 | 0 | 22 | 23 |
| *Proteus mirabilis* | 0 | 0 | 1 | 1 |
| **TOTAL** | **60** | **64** | **82** | **206** |
| **No bacterial growth** | **50** | **43** | **35** | **128** |

MC: MacConkey
